# Isolated and early-onset cerebral fat embolism syndrome in a multiply injured patient: a rare case

**DOI:** 10.1186/s12891-019-2736-4

**Published:** 2019-08-17

**Authors:** Chin-Kai Huang, Chih-Yuan Huang, Chia-Lung Li, Jui-Ming Yang, Chin-Hsien Wu, Chih-Hui Chen, Po-Ting Wu

**Affiliations:** 1 Department of Orthopedics, National Cheng Kung University Hospital, College of Medicine, National Cheng Kung University, Tainan, Taiwan; 2 Department of Surgery, National Cheng Kung University Hospital, College of Medicine, National Cheng Kung University, Tainan, Taiwan; 3 Division of Traumatology, Department of Surgery, National Cheng Kung University Hospital, College of Medicine, National Cheng Kung University, Tainan, Taiwan; 4 Department of Orthopedics, Tainan ShinLau Christian Hospital, Tainan, Taiwan; 5 Department of Biomedical Engineering, National Cheng Kung University, Tainan, Taiwan; 6 Department of Orthopedics, E-Da Hospital/I-Shou University, Kaohsiung, Taiwan; 7 Department of Orthopedics, Taichung Veterans General Hospital, 1650 Boulevard Sect. 4, Taichung, Taiwan; 8 School of Medicine, National Yang-Ming University, Taipei, Taiwan; 9 Department of Orthopedics, College of Medicine, National Cheng Kung University, 1 University Road, Tainan, Taiwan; 10 Department of Orthopedics, National Cheng Kung University Hospital Dou-Liou branch, College of Medicine, National Cheng Kung University, YunLin, Taiwan

**Keywords:** Cerebral fat embolism syndrome, Cognitive deficit, Long-bone fracture, Outcome

## Abstract

**Background:**

Fat embolism syndrome (FES) is a rare complication that can occur between 12 and 72 h after the initial insult. Isolated cerebral FES without pulmonary symptoms is rarer. Early fracture fixation might prevent FES. We report a case of multiple-fracture with FES despite definite fixation three hours post-injury.

**Case presentation:**

A 54-year-old man presented with multiple fractures: left femoral shaft (AO B2), left distal radius (AO C3), left comminuted patella, right comminuted 1st metatarsal base and left 2nd-4th metatarsal neck. Because he was stable, we gave him early total care and definite fixation, which required seven hours and yielded no complications. After he recovered from anesthesia, however, his eyes deviated right, his right upper arm was paralyzed, his consciousness level was poor, and his Glasgow Coma Scale score was E3VeM4. Chest X-rays showed clear lung fields, and brain computed tomography showed no intracranial hemorrhage. He did, however, have tachycardia, anemia, and thrombocytopenia. Brain magnetic resonance images showed a hyperintensive starfield pattern on diffuse weighted images, which suggested cerebral FES. After supportive care, his consciousness cleared on postoperative day 17, and he recovered full right upper arm muscle power after four months; however, he had a significant cognitive deficit. One-year post-injury, after regular rehabilitation therapy, he was able to independently perform his activities of daily living but still had a residual mild cognitive deficit.

**Conclusion:**

Early fixation can attenuate but not eliminate the incidence of FES. Early assessment and rehabilitation therapy might be required for patients with cerebral FES and cognitive deficits; however, such deficits are difficult to predict and need long-term follow-ups.

## Background

Fat embolism syndrome (FES) is a rare complication with a triad of symptoms: progressive respiratory distress, deteriorating mental status, and petechial rash. FES occurs between 12 and 72 h after the initial insult, and, in Taiwan, at a mean of 48.5 h after long-bone fractures [1]. According to the literature [2], the incidence ranges from < 1% to > 30% of cases. This wide variation in the incidence rate might be attributable to heterogeneous diagnosis criteria. The most frequently used criteria are Gurd’s [3] and Gurd and Wilson’s [4], which require that a diagnosis of FES meet at least one major criterion and at least four minor criteria. Seventy-five percent of patients with FES had respiratory dysfunction because of tachypnea, hypoxemia, or respiratory failure [1], and 86% had cerebral changes [5]. However, a pure central nervous system presentation without pulmonary symptoms is rare [1]. We report a rare case with multiple fractures followed by isolated cerebral FES, despite the patient’s having undergone early (three-hours post-insult) total care with definite fixation.

## Case report

**Fig. 1:**
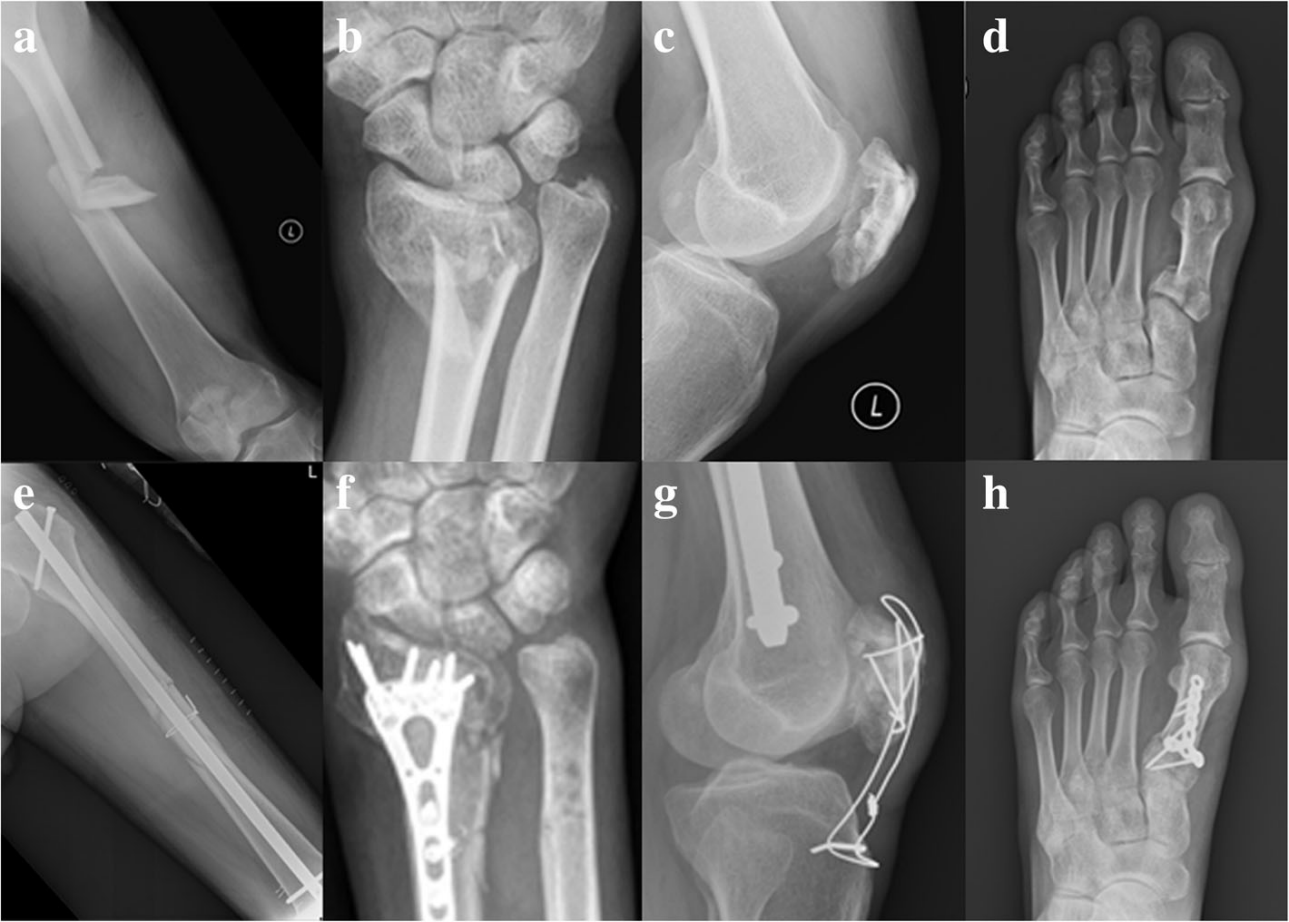
Fractures before and after surgery. **a** Left femoral shaft (AO B2). **b** Left distal radius (AO C3). **c** Left comminuted patella. **d** Right comminuted 1st metatarsal base. **e** Closed reduction and internal fixation with a reamed antegrade interlocking nail for the left femur. **f** Open reduction and internal fixation with plates for the left distal radius. **g** Interfragment Kirschner wires and patellotibial figure-of-eight wiring for the left patella. **h** Open reduction and internal fixation with plates for the right 1st metatarsal base

**Fig. 2:**
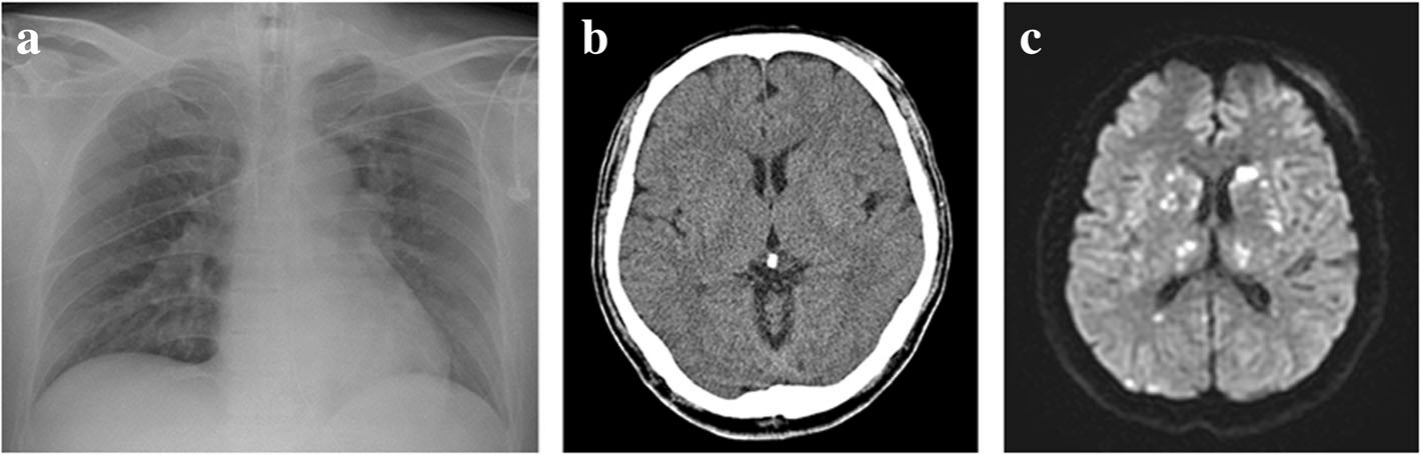
**a** A chest X-ray of a clear lung field. **b** A brain CT showing no intracranial hemorrhage. **c** A brain MRI showing multiple nodular or punctate hyperintensity lesions on diffusion-weighted (DW) images of the bilateral thalami, basal ganglia, cerebellar, and cerebral hemispheres

A 54-year-old man with no known underlying disease fell 4 m and was brought to the National Cheng Kung Hospital (NCKUH) emergency room. He had multiple fractures: left femoral shaft (AO B2), left distal radius (AO C3), left comminuted patella, right comminuted 1st metatarsal base (Fig. 1a-d), and left 2nd-4th metatarsal neck. A whole-body computed tomography (CT) scan showed no other associated injuries. About 3 h post-injury, he was sent to our operating room (OR) with a clear consciousness and 98–100% of peripheral oxygenation saturation in a normal indoor room atmosphere. Because he was physiologically stable, he was given early total care and a definite fracture fixation. Surgery lasted 7 h and yielded no complications. Closed reduction (CR) and internal fixation with a reamed antegrade interlocking nail for the left femur, open reduction and internal fixation with plates for the left distal radius and right 1st metatarsal base, and CR and fixation with Kirschner wires for the left 2nd-4th metatarsal neck were fixed in that order. Because the articular fracture of his left patella was unrepairable, interfragment Kirschner wires and patellotibial figure-of-eight wiring were used to restore the extensor mechanism. We found neither hypotension nor hypoxia during the surgery; postoperatively, however, the patient’s eyes deviated to the right side, his right upper arm muscle was paralyzed, and his Glasgow Coma Scale (GCS) score was E3V_E_M4 when he recovered from the anesthesia. A chest X-ray (Fig. 2a) shows a clear lung field, and a brain CT (Fig. 2b) shows no intracranial hemorrhage. Despite the patient’s low oxygen demand and no clinical evidence of acute respiratory distress syndrome (FiO_2_: 30%; PaO_2_: 108.6 mmHg; FiO_2_/PaO_2_ > 300), his endotracheal tube was retained for airway protection. On postoperative day (POD) 0, the patient had tachycardia (heart rate: 110/min), anemia (hemoglobin: 10.3 g/dL), and thrombocytopenia (platelet count: 131 × 10^3^/μL). He met only 1 major and 3 minor criteria. However, brain magnetic resonance image (MRI) (Fig. 2c) shows multiple nodular or punctate hyperintensity lesions on diffusion-weighted (DW) images of the bilateral thalami, basal ganglia, cerebellar, and cerebral hemispheres. We highly suspected FES and we gave the patient supportive treatment with additional methylprednisolone (Solu-Medrol™) and albumin. His GCS score fell to E2V_E_M4 on POD 4 and persisted at that level for the next 10 days. After POD 15, his consciousness improved and he was alert on POD 17, but his right upper arm muscle power score was 0 and left upper arm muscle power score was 3. On POD 29, his upper arm muscle power score was 3 on both sides. He could speak using only simple words instead of sentences, and he responded slowly. Four months post-injury, a complete cognitive test—the cognitive ability screening instrument (CASI) and the mini-mental status examination (MMSE)—showed significant deficits in recent memory, orientation, abstract thinking, and verbal fluency. The patient’s right upper and lower limb muscle power had fully recovered, however. Because of his cognitive impairment and left limb muscle atrophy, he underwent physical and cognitive rehabilitation. All fractures achieved union four months post-surgery (Fig. 1e-h). One-year post-injury, the patient’s left lower limb muscle power score had not fully recovered from left symptomatic traumatic patellofemoral joint arthritis. The muscle power scores of the other three limbs were full (five), and he was able to independently perform his activities of daily living. However, he continued to have a residual mild cognitive impairment.

## Discussion and conclusion

As in most other case reports and series on FES [6], fat and medullary content flowed into our patient’s bloodstream. However, FES has been reported in only 0.5–11% of patients with long-bone fractures [1], which might indicate that fat emboli are not significant for cardiopulmonary dysfunction. The stress response mediated by catecholamine is thought to be another possible mechanism of FES [7]. Isolated cerebral FES with primary neurological deficits is rare: however, an incidence of 10% was reported in one study [4]. Most FES cases have occurred between 12 and 72 h post-injury [1, 8], but one case of early-onset FES (< 12 h post-insult) has been reported [9]. Patients with long-bone fractures and multiple fractures are at high risk for developing FES [2]. Early fixation can reduce the incidence of FES [10]. However, in our case, the patient underwent fixation 3 h post-injury but developed FES. Therefore, early fixation might attenuate but not eliminate FES.

The current diagnostic criteria of FES are based on clinical signs and symptoms; Gurd’s and Gurd and Wilson’s criteria are most frequently cited [2]. Our case met only one major criterion (conscious change) and three minor criteria (tachycardia, thrombocytopenia, and anemia), which does not fully support the diagnosis of FES. However, Gurd’s [3] and Gurd and Wilson’s [4] criteria were more favorable on pulmonary FES, and MRIs have shown their potential for diagnosing cerebral FES early. Multiple small nodular or hyperintense punctate lesions on the DW MRI, just like a starfield pattern of scattered bright spots on a dark background, is a typical finding for cerebral FES [11]. It is thought that scattered bright spots are pathognomonic of acute cerebral microinfarcts and that the hyperintense signal reflects foci of cytotoxic edema [11]. Therefore, researchers emphasize the importance of MRIs, and some even consider it a minor criterion of FES [12]. However, diffuse axonal injury (DAI) might also present the same starfield pattern on DW MRI even though most patients with DAI immediately present with an unclear consciousness after a head injury [13]. Our DW MRI finding was typical, and the clinical presentations met the rule of “one major criterion and four minor criteria” for diagnosing FES after we added the minor criterion of a DW MRI starfield pattern. Finally, we diagnosed our patient with cerebral FES after we had excluded other intracranial lesions.

The mortality of FES is about 5–15%, and pulmonary dysfunction is its primary cause [1]. Although some devastating cases of massive cerebral FES have been reported [14–16], the prognosis of cerebral FES is generally favorable, with only small cognitive deficits or nearly full recovery in most cases [1]. Normal muscle tone with an active deep tendon reflex and retention of appropriate responses to pain are thought to be good prognostic signs [1]. However, cognitive impairment and mental disorders have not often been discussed in the literature. Two exceptions to this have reported a detailed neuropsychological assessment of one patient [17] and the long-term cognitive outcomes of another [18] with cerebral FES.

Cerebral manifestations of FES are highly variable and nonspecific. Mild cognitive dysfunction might be difficult to identify and is often underestimated because cerebral FES patients might have comorbid post-traumatic stress. Detailed cognitive and neurological assessments might help identify subtle cognitive dysfunction and might be indicated for all cerebral FES patients. Moreover, early diagnosis and early intervention might contribute to better outcomes [19, 20]. Cerebral FES treatment is primarily supportive [21]. The importance of adequate oxygenation to prevent secondary brain injury is emphasized during recovery from cerebral FES [22].

We reported a rare case of early-onset and isolated cerebral FES in which the patient underwent surgery for his multiple fractures three hours post-trauma. Early fixation can attenuate but not eliminate the incidence of FES. MRIs have become more important for diagnosing FES. Better supportive care might lead to more favorable prognoses. Early cognitive assessment and rehabilitation therapy might be indicated for all cerebral FES patients with an apparent cognitive impairment; however, such impairments are difficult to predict and require long-term follow-ups.

## Data Availability

All data concerning the case are presented in the manuscript.

## Abbreviations

CASI: Cognitive ability screening instrument
CR: Closed reduction
CT: Computed tomography
DAI: Diffuse axonal injury.
DW: Diffusion-weighted
FES: Fat embolism syndrome
GCS: Glasgow Coma Scale
MMSE: Mini-Mental State Examination
MRI: Magnetic resonance image
